# Coupled variability in primary sensory areas and the hippocampus during spontaneous activity

**DOI:** 10.1038/srep46077

**Published:** 2017-04-10

**Authors:** Nivaldo A. P. de Vasconcelos, Carina Soares-Cunha, Ana João Rodrigues, Sidarta Ribeiro, Nuno Sousa

**Affiliations:** 1 Life and Health Sciences Research Institute (ICVS), School of Medicine, University of Minho, Braga, 4710-057, Portugal; 2 ICVS/3B’s - PT Government Associate Laboratory, Braga/Guimarães, Portugal; 3 Brain Institute, Federal University of Rio Grande do Norte (UFRN), Natal, RN,59056-450, Brazil

## Abstract

The cerebral cortex is an anatomically divided and functionally specialized structure. It includes distinct areas, which work on different states over time. The structural features of spiking activity in sensory cortices have been characterized during spontaneous and evoked activity. However, the coordination among cortical and sub-cortical neurons during spontaneous activity across different states remains poorly characterized. We addressed this issue by studying the temporal coupling of spiking variability recorded from primary sensory cortices and hippocampus of anesthetized or freely behaving rats. During spontaneous activity, spiking variability was highly correlated across primary cortical sensory areas at both small and large spatial scales, whereas the cortico-hippocampal correlation was modest. This general pattern of spiking variability was observed under urethane anesthesia, as well as during waking, slow-wave sleep and rapid-eye-movement sleep, and was unchanged by novel stimulation. These results support the notion that primary sensory areas are strongly coupled during spontaneous activity.

The primary sensory cortex plays a critical role in the response to sensory stimulation from the environment, whereas the hippocampus (HP) is mostly implicated in memory. The technological developments in simultaneous recordings of large neuronal populations^1^,^2^,^3^,^4^,^5^ enabled a better understanding of the fundamental properties of their dynamics; particularly, those developments that boost our comprehension regarding spiking variability in large neuronal cortical populations, including amongst primary cortical sensory areas, and even during periods in the absence of stimuli, also called spontaneous activity, which seems to resemble properties of spiking-evoked activity in freely moving animals, in both the sensory cortex^6^,^7^,^8^ and the hippocampus^9^. The primary sensory cortices display different levels of variability in neuronal activity^10^,^11^,^12^,^13^, with behavioral relevance^14^,^15^,^16^,^17^,^18^,^19^, and specific relationship to different cortical states^16^,^17^,^20^,^21^,^22^. The variability level seems to be a proxy to the cortical state, from the macro to microscopic spatial scale, in a time resolution of seconds. Thus, it is of relevance to define the local network state as a measure of the level the spiking variability in a given neuronal population, rather than use, for instance, the N-letter binary word like to represent the instantaneous local network states^23^.

At the neuronal level, recent studies on single sensory pathways have shown that a large part of spiking variability originates from neuronal excitability fluctuations, which are correlated throughout time and among neurons^13^,^24^. Thus, a significant part of the cortical state seems to be coupled by the joint activation of sensory pathways during evoked responses. The neuromodulatory systems are also important sources of shared neuronal excitability fluctuations, most notably the cholinergic-driven^25^. Despite these advances, it is still not clear how neocortical circuits coordinate their instantaneous local neuronal network states at different spatial scales^23^,^26^,^27^,^28^. This information is still lacking not only for interactions between primary sensory cortices, but also between these and the hippocampus.

To address this issue, we used simultaneous recordings of the spontaneous activity of local neuronal populations in the primary sensory cortices and hippocampus. First, large-scale recordings from six local neuronal populations located 200 μm apart from each other were used along an anterior posterior path, at deep layers of the primary visual cortex in urethane-anesthetized rats. Then, to address the question on a large spatial scale, we investigated the activity of local neuronal populations recorded from deep layers of the visual and somatosensory primary sensory cortices, as well as from the hippocampus, in freely moving rats. The local network state was defined by the coefficient of variation (CV) of its spiking activity during the time period of a few seconds (e.g., 10 s)^20^,^29^. Finally, we evaluated whether the correlation level in the instantaneous local network states is affected by exposure to a novel multisensory experience^30^.

Strong correlation was found between local network states across local neuronal populations in primary sensory cortices, at both small and large spatial scales. Whereas, modest correlations were found between those areas and the hippocampus, despite a near zero spiking correlation structure^29^,^31^ between neuronal pairs significantly apart in both cross-modal cortico-cortical and cortico-hippocampal pairs. The current results show that local neuronal networks in primary sensory cortices change their spiking variability in a strongly correlated manner over long periods of time, at both small and large spatial scales. Cortico-hippocampal changes, however, are only moderately correlated across the several states investigated.

## Results

### Looking at a small spatial scale

**Figure 1:**
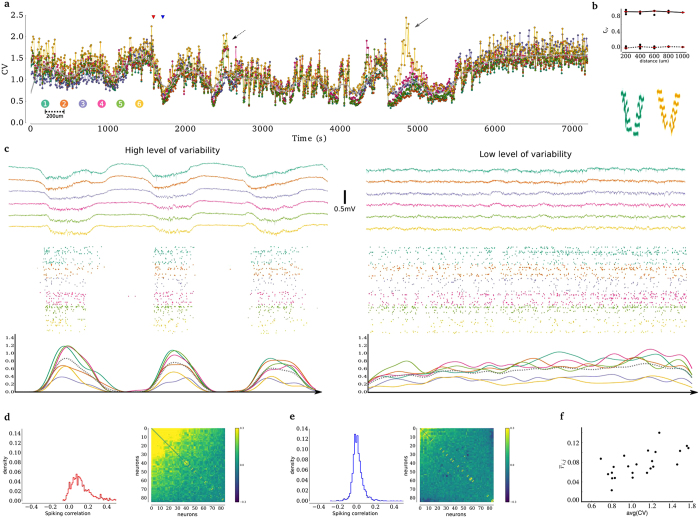
Coupling of the variability of simultaneous recordings across V1’s local neuronal populations in urethane-anesthetized rats during spontaneous activity. (**a**) CV within 10 s long independent blocks in which each dot represents one block throughout 2 h of recordings; each trace represents one local neuronal population (spot), one color per spot, gray continuous line represents the average curve. Triangles (red/blue) indicate the beginning of the time period used to sample inactive/active periods, respectively, in (b); arrows highlight time periods of reduced coupling. Dashed arrows represent 3 out of 6 spots that have higher variability than the other 3 spots; continuous arrow represents 1 out of 6 spots. (**b**) (top) Pearson’s correlation coefficient between the CVs as a function of the distance between spots; throughout the whole experiment, the red line indicates the median of the corresponding group; all p-values ≪ 0.001. Dashed line represents the same measure for Poisson-equivalent spike data with p-values ≫ 0.05 when compared to zero (except between spots 4 and 5). (bottom) Traces of action potentials of a spike train across the geometry used for recording channels in each cortical spot. (**c**) Samples of 4 s long recordings when inactive/active (left/right): (top) traces of raw data simultaneously recorded from six different shanks, one per spot approximately 200 μm apart, each one composed of 10 channels and colored according to the color code found in (a); (middle) corresponding raster plots for spikes found in each spot, one row per unit (n = 235), using the same color code found in (a), where MUA (n = 145)/SUA (n = 90) are surrounded by white/gray backgrounds, respectively; (bottom) corresponding population firing rate convolved with a Gaussian kernel, sigma = 100 ms, colored according the color code found in the raster plot, gray dashed line represents the mean population firing rate and vertical bars represent 1.5 spike/s/unit. (**d,e**) Spiking correlation for high/low levels of variability: (left) histogram and (right) correlation matrix with colormap in [−0.3;0.3]. (**f**) Scatter plot of mean CV versus mean spiking correlation in independent 300 s long periods.

In primary sensory cortical areas, neuronal coordination is estimated by the significant tails of the spike correlations. It has been shown that these significant tails decrease faster within 1000 μm of a rat’s primary cortex than when observed across nearby spots 200 μm horizontally apart in the same layer^29^. In addition to this apparent horizontal lack of coordination in relatively small spatial scale, our results suggest consistent ties among their local dynamics over long periods of spontaneous activity (>3600 s). A strong variability correlation was found during spontaneous activity between separate (hundreds of μm apart) primary visual cortex spots (V1), such as is illustrated in Fig. 1a and detailed in 1b according to the sites’ geometry.

Figure 1a shows the CV during spontaneous activity in the rat’s primary visual cortex, sampled in intervals of 200 μm along a line parallel to the antero-posterior axis (see Materials and Methods for details). A correlated spiking variability level across pairs of local neuronal networks was observed throughout the experiments (>3600 s; more examples in Fig. S1). The decrease in the amount of spiking correlation between neocortical neuronal pairs as the distance between those neurons increases throughout distances of 200 and 1000 μm is well known^29^,^32^. In addition, Fig. 1b (top) shows strong, stable and significant (all p-values < 0.01) correlated spiking variability levels in the primary visual cortex^23^. By contrast, the correlated spiking variability was not significant when all spike times were replaced by the corresponding Poisson’s equivalent spike trains (identical firing rates; all p-values > 0.1). Moreover, the occasional spiking variability decoupling among neuronal populations (arrows in Fig. 1a) shows that those high levels of variability coupling may sporadically drop down (quantification in Fig. 1a and in Fig. S2). Figure 1c illustrates the underlying neuronal activity across the primary cortical populations during spontaneous activity during periods of high and low levels of variability for the (top) wideband local field potential, (middle) raw spiking activity, and (bottom) population firing rate. Different levels of spiking variability imply spiking correlation structure. Panels d and e in Fig. 1 show them calculated during two 300 s long periods of high and low levels of variability, respectively. This is confirmed in the scatter plot of the average CV versus the mean spiking correlation within independent 300 s long periods throughout the whole experiment (Fig. 1f; adjusted R^2^ = 0.94, n = 23).

In summary, high levels of variability are related to a positive mean reflected in “hot” correlation matrices, whereas low levels of variability are related to near-zero mean correlation reflected in “cold” correlation matrices. Altogether, these data illustrate that different levels of spiking variability in the local neuronal networks in the primary visual cortex are related to different population spiking modes (spiking correlation structures)^17^,^20^,^22^. Furthermore, the high levels of similarity between spiking variability levels suggest that the population spiking modes are temporally shared among local neuronal populations during spontaneous activity, at least on small spatial scales.

### Looking at a large spatial scale

**Figure 2:**
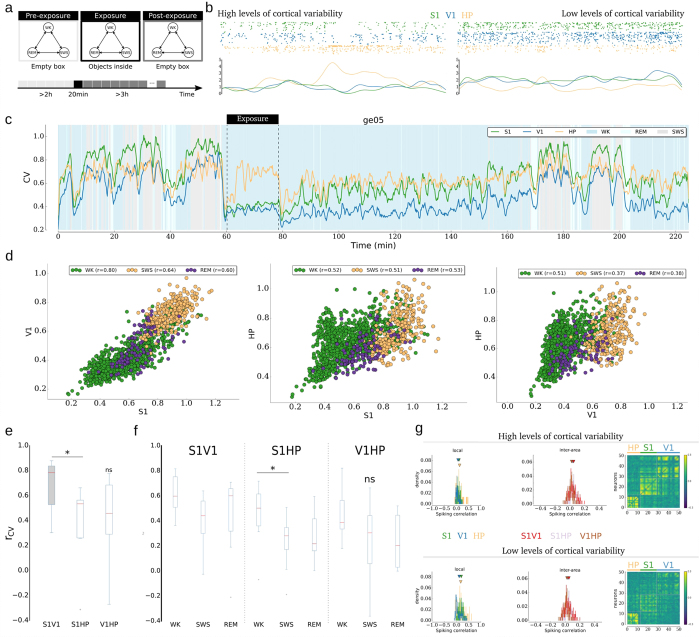
Coupling variability across primary sensory areas and HP. (**a**) Overall experimental design with freely behaving rats. (**b**) (top) Two seconds long samples of raster plots in time intervals with high and low cortical variability during spontaneous pre-exposure. (bottom) Corresponding 2 s long population firing rate plots of the raster plots found on (top) with the same color code after a 100 ms wide Gaussian kernel; the vertical bar corresponds to spikes/neuron/second. (**c**) Example of the CV of the population activity in each brain area: in S1 (green, n = 16), V1 (blue, n = 22) and HP (orange, n = 13) across different behavioral states; vertical black dashed lines delimit the exposure time period. The shading color code informs the behavioral states throughout the experiment, where each data point was calculated from 10s-long periods of population activity. (**d**) Scatter plots of pairs of CVs in each brain area shown in (**a**) segmented by selected behavioral states (WK,SWS and REM); also indicated is the Pearson correlation coefficient, r, between the CVs in each behavioral state. (**e**) Boxplot of group data for the Pearson correlation coefficient between CVs found in each pair of brain area during all experiments (8 datasets); all pairs had a significant correlation in CV with a significant difference only between S1V1 and S1HP. (**f**) Example of CV of the population activity segmented by behavioral states. (**g**) Samples of spiking correlations for 5 min long pre-experience time periods with different levels of cortical variability: (left) local spiking correlation histograms within S1, V1 and HP; (left-top) mean spike correlations for high levels of cortical variability; (left-bottom) mean spike correlations for low levels of cortical variability; (middle) inter-area spiking correlation histograms; (middle-top) mean spike correlations for low levels of cortical variability; (middle-bottom) mean spike correlations for low levels of cortical variability; (right, top and bottom) respective correlation matrices of the data.

Similar to what was observed in urethane-anesthetized rats (Fig. 1), freely behaving rats showed different levels of cortical variability in spontaneous activity associated with different population spiking modes (Fig. 2b, top). Namely, high levels of cortical variability were associated with a high density of populational silences, whereas low levels of cortical variability were associated with a sparse occurrence of populational silences. Additionally, there were large (small) fluctuations in population firing rate (Fig. 2b, bottom) during periods of high (low) levels of variability. Analysis of the CV for S1, V1 and HP during the experimental session reveals a strong Pearson correlation of the CV (normally distributed, p < 0.05) from distinct cortical areas (r_S1V1_ = 0.8776). Conversely, there was only a modest correlation between HP and either primary sensory areas S1 or V1 (r_S1HP_ = 0.5270 and r_V1HP_ = 0.4826; both p ≪ 0.01, Fig. 2c). Despite the high level of correlation between instantaneous local neocortical network states, levels were clearly different, which suggests that they do not necessarily share levels of variability and their spiking modes but rather share the timing and direction of changes of their local neuronal network states (Fig. 2c and Fig. S1).

**Figure 3:**
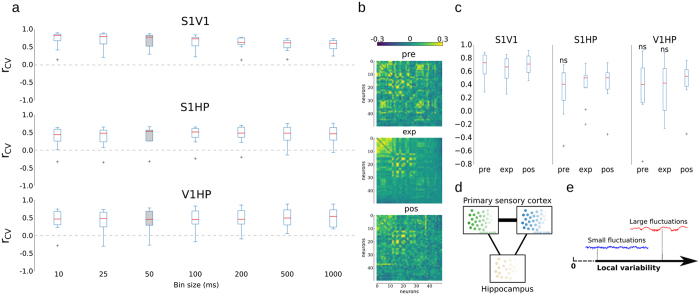
Time-scale and influence of novelty in coupling variability across brain areas. (**a**) Group data (n = 8): coupling on global variability across different time scales. On the vertical axis is shown the correlation between the coefficient of variation of population rate found in primary sensory cortices: S1, V1, and HP, and on the horizontal axis is shown the different bin sizes for the calculated coefficients of variation, one per pair of area/animal. Highlighted is the default bin size used in the main text (50 ms). The median of rCV significantly differs from zero (p < 0.01) in all conditions. (**b**) Samples of spiking correlation matrices based on the activity found in HP, S1 and V1 during (left) pre-exposure, (middle) exposure, and (right) post-exposure to novel objects, sorted by loadings of first principal component calculated based on correlated spiking activity during exposure. (**c**) Group data (n = 8) of Pearson’s correlation among the coefficient of variation of neuronal population activity found in S1, V1 and HP during pre-exposure (pre), exposure (exp) and post-exposure (post) to novel objects. (**d**) General proposal for coupling in variability in cortical activity. At small spatial scales of local cortical neuronal populations in the primary sensory cortex and hippocampus, local neuronal populations (circles) show strong shared variability and share very similar levels of variability across time, whereas at large scales, the primary sensory areas do not necessarily share their level of variability (spiking mode) but strongly share timing when the variability changes This last type of shared variability is only modest between cortex and hippocampus. (**e**) Illustration of the variability axis.

There is robust evidence regarding changes in cortical variability across different behavioral states^10^,^16^,^18^,^33^. However, the temporal coupling in variability, and in cortico-cortical and cortico-hippocampal activity, during different behavioral states remains unknown. Thus, segmentation of the data according to the three major behavioral states studied was also performed: waking (WK), slow wave sleep (SWS), and rapid eye movement sleep (REM)^16^. This analysis indicated that the cortical spiking activity during wakefulness had significantly higher variability coupling than that observed during both sleep states (Fig. 2d; p ≪ 0.01). In addition, cortico-hippocampal coupling (both S1-HP and V1-HP) was also smaller than that observed between cortical areas. Importantly, the group data analysis (total of single units isolated 685, #S1 = 209, #V1 = 227, and #HP = 249; see Table S1) showed that coupling among primary sensory areas (S1V1) was significant (Fig. 2e; Wilcoxon, p_S1V1_ = 0.012, p_S1HP_ = 0.036 and p_V1HP_ = 0.025), and higher (Fig. 2e; Kruskal-Wallis, p = 0.036) than the coupling between primary sensory areas and HP for longer periods of time (Table S1), across different time-scales (Kruskal-Wallis p < 0.01; Fig. 3a). There was also significant coupling among the brain areas studied (Fig. 2f) across behavioral states (Wilcoxon, p < 0.05), except for V1HP during SWS (p = 0.067). Analysis of the same brain area pair across behavioral states (#animals: n_WK_ = 8, n_SWS_ = 8, and n_REM_ = 7), demonstrated a significant difference only in S1-HP between WK and SWS (p = 0.046) and a trend in S1-V1 between WK and SWS states (p = 0.115).

When analyzing the properties of spiking correlations calculated in periods of 5 min of high and low levels of variability in spontaneous activity during the pre-experience interval (n_HP_ = 78, n_S1_ = 120 and n_V1_ = 231; n_S1V1_ = 216, n_S1HP_ = 117 and n_V1HP_ = 195), it was confirmed that high levels of cortical variability were related to positive mean correlation structure, *r*, in cortical populations, but also in the HP (Fig. 2g, left-top; r_S1_ = 0.1817, r_V1_ = 0.0907, and r_HP_ = 0.1629, all p ≪ 0.01). By contrast, during periods of low levels of correlation of cortical variability, there was a near-zero mean correlation structure in cortical populations (r_S1_ = 0.0844, r_V1_ = 0.0404, and r_HP_ = 0.1119, all p ≪ 0.01) but still a positive correlation in the HP (Fig. 2g, left bottom panel). These results are supported by the average CV versus the mean local spiking correlation within independent 300 s long periods (Fig. S6). The coupling among these brain areas seems to be, on average, non-correlated regardless of the cortical levels of variability, as illustrated in Fig. 2g (middle panels): for a high level of cortical variability (Fig. 2g, middle-top), r_S1V1_ = 0.0374, r_S1HP_ = 0.0374, and r_V1HP_ = 0.0116 (all p ≪ 0.01); and for a low level of cortical variability (Fig. 2g, middle-bottom), r_S1V1_ = 0.0179, r_S1HP_ = 0.0394, and r_V1HP_ = 0.0050 (all p ≪ 0.01, except for V1-HP where p = 0.0285). The same is also shown in intra-region correlation areas in matrices correlation, for both conditions (Fig. 2g right panels, top and bottom).

### Novelty does not trigger significant changes in the variability structure

Here, data confirmed that spontaneous activity recorded during pre- and post-stimulation have similarities in spiking patterns, as previously shown in both the primary sensory cortex^7^,^34^ and HP^35^ (Fig. 3b–d). This has been shown in a previous study using this freely moving dataset, which proves that the exposure to novel objects promotes a clear change in the firing rate levels, mostly during SWS in S1, which lasts for hours^30^. However, it is still not clear how these changes impact variability coupling during spontaneous activity. In fact, Fig. 3b shows a striking similarity of the correlation matrix calculated based on a whole experiment with the correlation matrix based on a 100 s long period after the exploration of novel objects. However, we could not observe any significant changes in the level of correlation between the instantaneous variability in local neuronal networks when we segmented the data according to the exposure timing: pre-exposure, exposure, and post-exposure (Fig. 3c). This negative result suggests that the exposure to novel stimuli (in the dark) does not significantly change the local neuronal network coupling of instantaneous variability at deep layers of primary sensory cortices or between local cortical and hippocampal networks.

## Discussion

Current knowledge on correlated variability at the large neuronal population level in the primary sensory cortex is mostly based on evoked activity^8^,^13^,^21^,^22^. There are no studies addressing the issue of shared variability in spontaneous spiking activity from the non-local neuronal populations. Here we show that the variability in spontaneous activity is shared at both small and large spatial scales in the primary sensory cortices, revealing highly synchronized changes in variability across time in these areas. At small spatial scales, the variability, besides being temporally correlated, seems to display similar values. This suggests that at small spatial scales the local neuronal populations in the primary sensory cortex have similar dynamics during spontaneous activity, whereas at large-spatial scales, the local neuronal populations only share temporal dynamics. In fact, there seems to be an axis of the level of shared variability during spontaneous activity: from high levels in which the neuronal populations display similar levels of variability across time to low levels in which only the changes in the levels of variability are correlated over time despite having distinct values (Fig. 3d,e). The potential influences of anesthesia in coupled variability might be considered when comparing data among anesthetized and freely moving experiments.

The current findings shed light on a specific property of the dialogues within the primary sensory cortical areas and between the primary sensory cortices and hippocampus. Therefore, they expand our knowledge regarding intra-are and inter-area coupling in the brain^36^,^37^,^38^,^39^. The findings lead us to consider each local neuronal population in deep layers of the rat’s primary sensory cortex as a computational unit within a large primary sensory neocortical network, coordinated to perform information processing beyond local circuits, within and among “modality structured” primary sensory areas^40^.

The mechanisms underlying shared variability across primary sensory cortices during spontaneous activity are still unclear; amongst several options, variations in modulatory neurotransmitter systems are the best candidates^41^. More specifically, cholinergic inputs from nucleus basalis can exert a profound effect on cortical variability^14^,^17^,^42^,^43^. Thus, we hypothesize that the higher the level of shared inputs from neuromodulatory projections, the higher the level of shared variability by a pair of local neuronal populations from the primary sensory cortices or hippocampus.

## Methods

*In vivo* experiments, which target recordings from the primary visual cortex (V1, Bregma: AP = −7.2, ML = 3.5) used urethane- anesthetized rats (n = 5): Wistar Han, male, 350–500 g, 3–6 months old, 1.44 g/kg urethane. The stereotaxic coordinates for craniotomy and probe insertion in V1 combined information from Paxinos Atlas^44^ and recent studies^30^,^45^,^46^,^47^. The 64-channel silicon probe was then inserted around the central coordinate along the direction defined by the AP axis. The experimental protocols for *in vivo* experiments (housing, surgery and recordings) were performed according to FELASA guidelines^48^ and also in accordance with European Regulations (European Union Directive 2010/63/EU). Animal facilities and the people directly involved in animal experiments were certified by the Portuguese regulatory entity – DGAV (*Direcção-Geral de Alimentação e Veterinária*). All protocols were approved by the Ethics Committee of the Life and Health Sciences Research Institute (ICVS).

The data recorded from freely behaving animals have been extensively described in previous publications^30^,^45^,^46^,^47^. Thus, a brief description of the data is provided here. In total, electrophysiological data from 17 adult male rats (Long-Evans, 300–350 g) were evaluated, but only a subset (n = 8) satisfied the data analysis requirements: minimum number 4 of single units per area, minimum baseline recording time of 2 h before exposure, and minimum total duration of distinct main behavioral states (WK and SWS, see Table S1). Thus, herein, only a brief description of the data will be provided. Multi-electrode arrays made of single tungsten wires were implanted under anesthesia using the following stereotaxic coordinates: hippocampus (HP; AP: −2.80; ML: +1.5; DV: −3.30), S1 (AP: −3.00; ML: +5.5; DV: −1.40), and V1 (AP: −7.30; ML: +4.00; DV: −1.30). DV measurements considered the pial surface as a reference to target layer 5. Arrays consisted of 16 or 32 micro-wires spaced with 250 μm intervals (4 × 4 arrays for S1 and V1, 2 × 16 array for HP) attached to plastic connectors (Omnetics, Minneapolis, MN). In HP, approximately 80% of the electrodes were targeted to CA1; thus, DG neurons were pooled with CA1 neurons in all analyses in this paper. The position of the electrodes was histologically confirmed^30^. After a rest period of 7 days, simultaneous neuronal recordings were performed in S1,V1 and the HP in freely behaving rats inside a dark empty box for a 2 h period, after which four novel objects were inserted (one in each box’s corner). Animals were allowed to freely explore the objects for 20 min, after which all objects were removed from the box, and recordings proceeded for >3 h, as is illustrated in Fig. 2a 30 (also see Table S1a and b). It was a software package for supervised real-time spike sorting (SortClient 2002, Plexon Inc, USA).

The data that support the findings of this study are available from the corresponding authors upon reasonable request. Please refer to the Supplementary information for more details about the experimental procedures.

## Additional Information

**How to cite this article:** de Vasconcelos, N. A. P. *et al*. Coupled variability in primary sensory areas and the hippocampus during spontaneous activity. *Sci. Rep.* **7**, 46077; doi: 10.1038/srep46077 (2017).

**Publisher's note:** Springer Nature remains neutral with regard to jurisdictional claims in published maps and institutional affiliations.

## Supplementary Material

Supplementary Information
