# Supplementary material for: Coupled variability in primary sensory areas and the hippocampus during spontaneous activity
Source: Sci Rep. 2017 Apr 10;7:46077. doi: 10.1038/srep46077 (PMC5385523; doi:10.1038/srep46077)
Supplement: Supplementary Information [file srep46077-s1.pdf]

Coupled variability in primary sensory areas and the hippocampus during spontaneous activity

Nivaldo A P de Vasconcelos[1,2], Carina Soares-Cunha[1,2], Ana João Rodrigues[1,2], Sidarta Ribeiro[3,*], and Nuno Sousa[1,2,*]

[1]Life and Health Sciences Research Institute (ICVS), School of Medicine, University of Minho, Braga, 4710-057, Portugal.
[2]ICVS/3B's - PT Government Associate Laboratory, Braga/Guimarães, Portugal.
[3]Brain Institute, Federal University of Rio Grande do Norte (UFRN), Natal, RN,59056-450, Brazil.

ABSTRACT

The following sections, provide supplementary descriptions of the data, and analyses employed. The first section contains detailed information regarding from anesthetized rat data. The second gives a brief description of the recordings from freely behaving rats, which have been used in previous publications where detailed methodological information is available[1]. The last section covers the methods used for data analysis, as well supplementary support for some statements of the manuscript.

Experiments in anaesthetized rats

Anaesthesia and surgery

In vivo experiments which targeted recordings from primary visual cortex (V1) used Wistar Han rats (n=5). Animal (male, 350-500g, 3–6 months old) received a subcutaneous (s.c.) saline injection (2 ml) to prevent dehydration and were then anaesthetized with 1.44g/Kg urethane (fresh 20% solution in saline), administered in 3 intra-peritoneal (i.p.) injections 15 min apart. Atropine methyl nitrate (0.05 mg/kg, i.p., Sigma) was used to mitigate respiratory complications during the surgery (Clement et al., 2008). In some experiments, urethane supplements were administered in order to reach proper levels of analgesia before the surgery. Lidocaine (4%, s.c.) was applied on top of the skull at the onset of the surgery. After analgesia was reached the skin was cut, cleaned and dried, and then the stereotaxic procedures began, as described in the following section. Glucose was administered (2ml, 5%, s.c.) to rehydrate and replenish internal glucose levels before insertion of the silicon probe. Housing, surgical, and recording procedures were in accordance with the Life and Health Sciences Research Institute guidelines and the Minho University Institutional Animal Care and Use Committee, in in accordance with European Regulations (European Union Directive 2010/63/EU).

Stereotaxic procedures

The stereotaxic coordinates for craniotomy and probe insertion in V1 combined information from the Paxinos Atlas(Paxinos, n.d.) and recent studies[2] and recent studies[1,3–5]. A craniotomy of 2.5mm diameter (2.5mm) was centered on the following coordinates (mm) from Bregma: AP=-7.2, ML=3.5. The probe was then inserted around the central coordinate, along the direction defined by the AP axis. The central position coordinates could be rescued, even after the craniotomy, based on additional landmarks made in the bone. The silicon probe (BuzsakiA64sp, Neuronexus) was slowly inserted (100 um/min) through the dura mater, orthogonal to the horizontal plane defined by the stereotaxic, up to a depth around 1.100 um from the *pia mater*. Before inserting the silicon probe, has been administered 2ml (s.c.) of glucose in order to rehydrate, and replace the internal glucose storage as well.

Electrophysiological recordings

The electrophysiological recordings has been done using a 64-channels silicon probe (BuzsakiA64sp, sharpened tips enabled, Neuronexus) with the following geometry: 64 sites in 6 shanks, 10 sites/shank tip; shanks were 200 um apart and each site's area was 160 um^2, with impedance of 1–3 Mohm at 1KHz, disposed from the tip in a staggered configuration, 20 um apart. The electrophysiological recordings has been done by combining a Intan headstage (RHD 2164) with a Open Ephys box, at 30KHz/channel 16bits/sample.The probe was inserted in V1, connected to a Neuronexus probe adapter, and then plugged by an Omnetics connector to a headstage (Intan, RHD 2164) which amplified and digitized the analog signals, which were then sent to an Open Ephys box at 30KHz/channel 16bits/sample. The data were accessible through software interface by Open Ephys

Project, which enabled online view of the electrophysiological recordings. The off-line raw extracellular recordings were stored (locally and at http://osf.io) for later processing, including automatic and manual spike sorting.

Spike sorting

Off-line spike sorting from raw extracellular recordings was performed with the software provided by Klusta-Team[6,7]. Since the shanks were >200um, the activity between channels in different shanks was considered independent for spike sorting purposes, therefore spike sorting was performed in parallel, one per shank. The independence among spike data from different shanks was confirmed by the coherence matrices based on segments of raw data after bandpass filtering 300–500 Hz, based on customized Matlab code from Kenneth Harris. Spike sorting began with spike detection (filtered from 500 Hz–11.5KHz, with a 3rd order Butterworth), followed by the automatic spike sorting using KlustaKwik[7], which is an "masked" expectation–maximization (EM) algorithm for automatic clustering action potential waveforms. The outcome of the automatic part of the spiking sorting is a set of clusters of waveforms and the correspondent values of quality for each cluster. Using the Klustaviewa[7] the set of clusters was refined by merging and splitting operations, and selection adopted the following criteria: (a) low contamination ($< 5\%$) within the refractory period (2ms); (b) spiking stability across the period of interest; (c) minimum firing rate (0.5Hz). The final outcome of the spike sorting procedure was a set of spike trains, in which the i-th spike train was defined by the timestamps of action potentials assigned to i-th neuron within a given neuronal population. The automatic part of the spike sorting was executed in local computers and also in a computer cluster provided by the Portuguese initiative for scientific grid computing (INCD/LIP).

Experiments in freely behaving animals

Behaviours were continuously recorded under visible or infrared light with a CCD camera connected to a video tracking system (Cineplex) synchronized to the neural recordings. Illumination in the visible spectrum was measured inside the recording chambers, and the absence of visual stimuli in the visible spectrum was verified using a Minipa Electronics luximeter, model MLM - 1011. Recordings of large neuronal populations were performed using a 96-channels Plexon recording system (MAP, Plexon Inc, Dallas, TX). The animal's behavioural states were classified based on a previously described method, based on hippocampal and cortical LFPs[8]. Briefly, a two-dimensional state space was defined by two spectral amplitude ratios calculated by dividing energy at selected frequency bands from LFPs simultaneously recorded in the areas of interest. The states were classified based on amplitudes ratios of the LFPs within selected bands.

Data analyses

We used the same approach used to asses the coefficient of variation in a previous study[9], which we will briefly describe herein. For our purpose, the spike train of a given single- or multiunit is represented by the ordered sequence of time-stamps $u_i = [t_i]$ in which were detected spikes from that single- or multi-unit. Given a set of N population spike trains $S = u_i$, the correspondent population activity spike train is represented for $\bigcup_{u_i \in S} u_i$. Some of a spike calculations has been done only over part of spike trains, therefore it would be convenient define a slice of a spike train u_k in a given time interval I as the ordered sequence $[u_k]_I = [t_k; t_k \in I]$ or just u_k^I. It also convenient to define the time-series based on single spike trains, and the most known is the spike count $z_k = h(u_k, \Delta t)$, which is given by the histogram h over a spike train uk using a bin size t; and similarly would be done over slice of spike trains $z_k^I = h(u_k^I, \Delta t)$; and even over the population activity in S, $z_S^I = h(u_S^I, \Delta t)$.

Coefficient of variation

The coefficient of variation, CV, is an example of calculation over slices of spike trains: the CV of the neuronal activity found in neuronal population, S, during a time interval I, in a given time scale, is given as ratio between the standard deviation and mean found in that spike count, such as is defined in Equation (1).

$$CV(u_S^I, \Delta t) = \frac{s(z_S^I)}{\mu(z_S^I)} \tag{1}$$

where z_S^I is the corresponding spike count for u_S^I using a time scale Δt. Herein, the default parameters to calculate CVs were $\Delta t = 50ms$, over consecutive non-overlapping 10s-long time periods. Whereas in Fig S2 were used different time-scales, such indicated in its horizontal axis.

Spiking correlations

Usually the spiking data can be modulated by exogenous signals, which would distort the estimation of spiking correlations, usually biased by slow fluctuations in exogenous signals[10]. To avoid this issue and to be able to specify, *a priori*, the time scale

for the spiking correlations, we took advantage of an approach applied in previous studies[9], which convolved a Mexican-hat kernel with a spike count version of raw data, such as is briefly described in this section.

Given a set of spike trains, $S = \{u_i\}$, and a corresponding set of spike count time-series $Z = \{z_i\}$ at millisecond resolution ($\Delta t = 1ms$). Before to quantify the spiking correlation is demanded use a time-filter in order to specify the time-scale in which the spiking correlation should be estimated by using a Mexican hat filter, which in its turns is derived from the difference between two Gaussians, with zero mean and different standard deviations, s_1 and s_2, whose define the time-scale of hat filter, $h_{s_1,s_2}(t)$. Therefore, given a time-scale and its corresponding Mexican hat filter, $h_{s_1,s_2}(t)$, the first step in order to quantify the spiking correlation is convolve it with each time-series in Z , and then produce an equivalent set of rate (discrete) functions $\{n_i(t)\}$, where the i-th rate function is given by the convolution of the filter $h(t)$ with i-th spike count time-series, $z_i(t)$, in Z, such as is defined in the Equation (2).

$$n_i(t) = h_{s_1,s_2}(t) * z_i(t) \tag{2}$$

where s_1 and s_2 are the different standard deviations used to build the Mexican-hat filter, with $s_1 > s_2$, which define the time-scale to be observed into the neuronal activity data; in the current data analysis we have used $s_2 = \sqrt{17}s_1$, with the following default values: $s_1 = 100ms$ and $s_2 = 400ms$.

Given the set of rate functions $F = \{n_i(t)\}$ the ultimate step was calculate the covariance matrix. The covariance between a pair of rate functions, n_i and n_j is defined by Equation (3).

$$\sigma_{i,j} = \frac{1}{L}\sum_{t=0}^{L-1}(n_i(t)-\mu_i)\cdot(n_j(t)-\mu_j) \tag{3}$$

where L is the (integer) number of milliseconds in each rate functions. Once the kernel used in Equation (2) produces zero mean rate functions, the Equation 3 conveniently becomes a dot product such as in Equation (4).

$$\sigma_{i,j} = \frac{1}{L}\sum_{t=0}^{L-1}(n_i(t)\cdot n_j(t)) \tag{4}$$

Given that each rate function is a finite discrete time function, with millisecond resolution, each one of those rate function can be represented as finite sequence, and based on those sequences we defined the rate matrix, $B = [n_{i,j}]_{N\times N}$, where each $n_{i,j}$ is the instantaneous firing rate of the **i**-th neuron on the j-th millisecond, N is the number of recorded neurons. Based on rate matrix B, the covariance matrix is given by the Equation (5).

$$C = [\sigma_{i,j}]_{N\times N} = B.B^T \tag{5}$$

The correlation matrix, $C = [r_{i,j}]_{N\times N}$ can be calculated from the covariance, where $r_{i,j} = \frac{\sigma_{i,j}}{\sigma_i\cdot\sigma_j}$, and s_k is the standard deviation of k-th rate function, n_k , and $\sigma_{i,j}$ is given the Equation (3). The eigenvectors of the covariance matrix, C, defined in Equation (5), are called the principal components (PC) of this rate data, and the coordinates of a given PC are called its the *coefficients* or *loadings*, which define the linear combination of the rate functions in the direction to that PC, for instance the first principal component (PC1) over time is given the Equation (6).

$$PC_1(t) = \sum_{j=1}^{N}\lambda_{i,j}\cdot n_j(t) \tag{6}$$

where $\lambda_{i,j}$ is the j-th loading of the PC1, and $n_j(t)$ is the j-th rate function such as has been defined in the Equation (2). In order to avoid sensitivity to heterogeneity in the individual variance usually found in neuronal populations, we used PC based on correlation matrices instead of based on covariance matrices[11].

Variability around the mean activity

Given that mean population activity in neuronal population S with N units is $\overline{n}(t) = \frac{1}{N}\sum_{i=1}^{N}n_i(t)$, and taking $\sigma_{\overline{n}}^2$ as its variance, from basic statistics:

$$\sigma_{\overline{n}}^2 = \frac{1}{N^2}\sum_{i=1}^{N}\sigma_i^2 + \frac{2}{N^2}\sum_{i\neq j}^{N}\sigma_{i,j}^2 \tag{7}$$

where σ_i^2 is variance of the i-th rate function, and $\sigma_{i,j}^2$ is the covariance between a pair of rate functions n_i, n_j. Given that the average covariance is $\overline{\sigma}_\times^2 = \frac{1}{N(N-1)}\sum_{i=1}^N \sigma_{i,j}$ the Equation (7) can be rewritten:

$$\sigma_{\overline{n}}^2 = \frac{1}{N^2}\sum_{i=1}^{N}\sigma_i^2 + \frac{2(N-1)}{N}\cdot\left(\frac{1}{N(N-1)}\sum_{i\neq j}^{N}\sigma_{i,j}^2\right) = \frac{1}{N^2}\sum_{i=1}^{N}\sigma_i^2 + \frac{2(N-1)}{N}.\overline{\sigma}_\times^2 \tag{8}$$

Therefore, when $N \gg 1$, $\sigma_{\overline{n}}^2 \approx \overline{\sigma}_\times^2$. Thereby, in a large neuronal network, the size of fluctuation over the mean activity is in the same order of the average covariance in that population. Therefore, just by looking at the level of variability around global fluctuation in local neuronal population is possible estimate the kind of spiking correlation structure can be found in that neuronal substrate, and consequently its dynamics: a synchronized neuronal activity is related to a covariance/correlation structure with positive mean, and thus large fluctuations around the mean activity; whereas desynchronized neuronal activity is related to a near zero covariance/correlation structure, and thus small fluctuations around the mean activity[9,12–15].

(a)

Dataset	S1	V1	HP	Total
ge03	23	38	4	65
ge04	13	28	4	45
ge05	16	22	13	51
ge06	29	7	22	58
ge12	33	40	89	162
ge13	31	27	38	96
ge14	25	23	34	82
ge17	39	42	45	126
**Total**	**258**	**313**	**327**	**898**

(b)

Dataset	WK	SWS	REM	WT	Total
ge03	7930	5530	1300	0	14760
ge04	8730	5180	440	2710	17060
ge05	9710	2730	1040	0	13480
ge06	5800	6000	1660	170	13630
ge12	5740	10680	1800	190	18410
ge13	10690	12390	60	2070	25210
ge14	8690	6380	0	50	15120
ge17	12280	8260	380	50	20970
**Total**	**69570**	**57150**	**6680**	**5240**	**138640**

Table S1. General information about the datasets. (a) Number of neurons/area in each dataset. (b) Sum of durations (in seconds) of the different behavioural states: WK, SWS, REM and WT; for each selected dataset.

References

1. Ribeiro, S. *et al.* Novel experience induces persistent sleep-dependent plasticity in the cortex but not in the hippocampus. *Front. Neurosci.* **1**, 43–55 (2007).
2. Paxinos, G. & Charles, W. *The Rat Brain in Stereotaxic Coordinates* (Elsevier, 2016).
3. Almeida-Filho, D. G. *et al.* An investigation of hebbian phase sequences as assembly graphs. *Front. Neural Circuits* **8**, 34 (2014).
4. Silva, B. *et al.* Statistical characterization of an ensemble of functional neural networks. *Eur. Phys. J. B* **85**, 1–9 (2012).
5. Vasconcelos, N. *et al.* Cross-modal responses in the primary visual cortex encode complex objects and correlate with tactile discrimination. *Proc. Natl. Acad. Sci. U. S. A.* **108**, 15408–15413 (2011).
6. Kadir, S. N., Goodman, D. F. M. & Harris, K. D. High-dimensional cluster analysis with the masked EM algorithm. *Neural Comput.* **26**, 2379–2394 (2014).
7. Rossant, C. *et al.* Spike sorting for large, dense electrode arrays. *Nat. Neurosci.* (2016).
8. Gervasoni, D. *et al.* Global forebrain dynamics predict rat behavioral states and their transitions. *J. Neurosci.* **24**, 11137–11147 (2004).
9. Renart, A. *et al.* The asynchronous state in cortical circuits. *Science* **327**, 587–590 (2010).
10. Shadlen, M. N. & Newsome, W. T. The variable discharge of cortical neurons: implications for connectivity, computation, and information coding. *J. Neurosci.* **18**, 3870–3896 (1998).
11. Jolliffe, I. T. *Principal Component Analysis*. Springer Series in Statistics (Springer New York, 2013).
12. Cohen, M. R. & Kohn, A. Measuring and interpreting neuronal correlations. *Nat. Neurosci.* **14**, 811–819 (2011).

13. Ecker, A. S. *et al.* Decorrelated neuronal firing in cortical microcircuits. *Science* **327**, 584–587 (2010).

14. Harris, K. D. & Thiele, A. Cortical state and attention. *Nat. Rev. Neurosci.* **12**, 509–523 (2011).

15. Schölvinck, M. L., Saleem, A. B., Benucci, A., Harris, K. D. & Carandini, M. Cortical state determines global variability and correlations in visual cortex. *J. Neurosci.* **35**, 170–178 (2015).

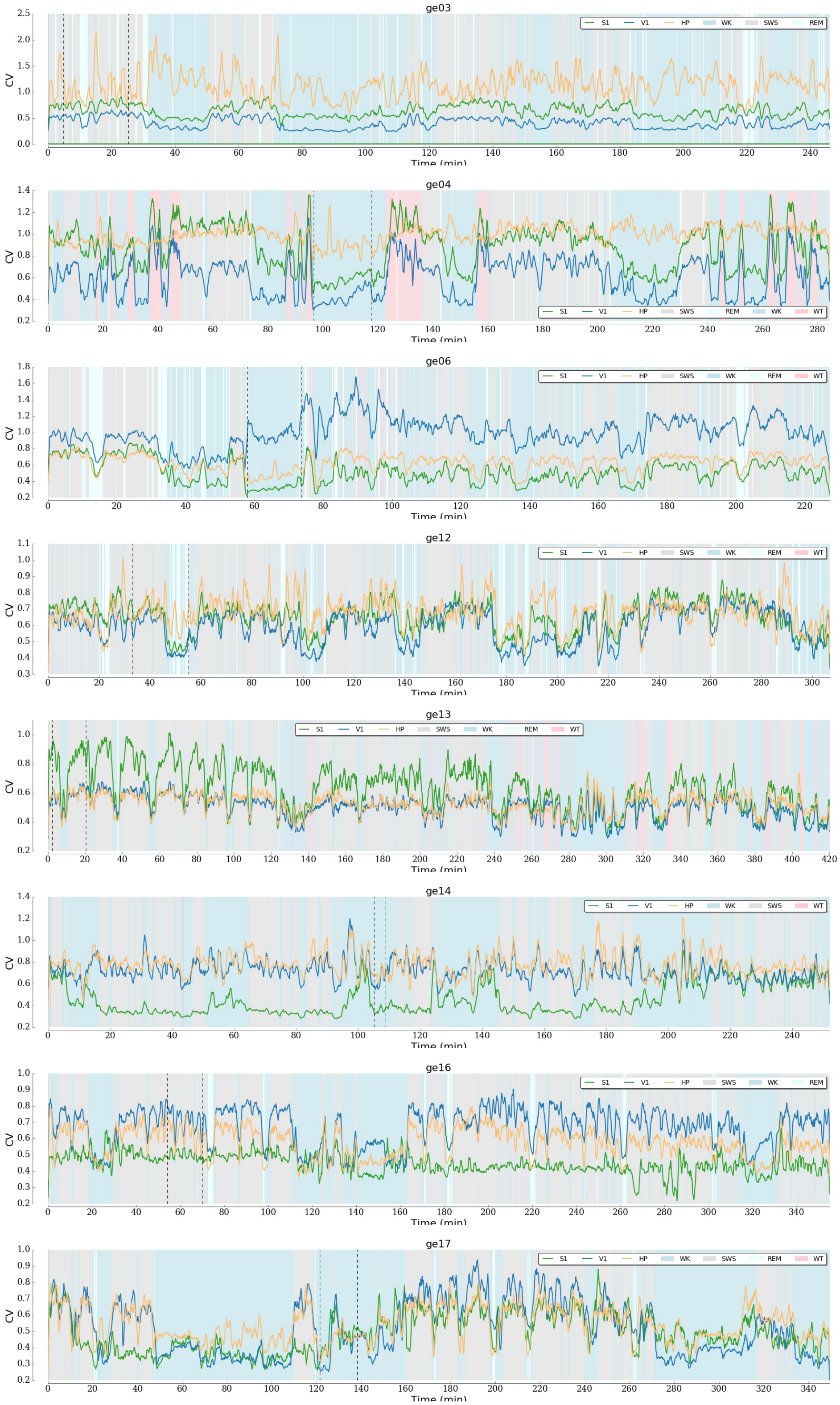

Figure S1. Coefficient of variation of population rate in primary sensory neocortex: somatosensory (S1), visual (V1); and hippocampus (HP), one per animal, where are represented up to four different behaviour states: wakefulness (WK), slow wave sleep (SWS), rapid eyes movement (REM) and whisker thinking (WT).Vertical black dashed lines delimit the time period when there was exposure to novel objects.

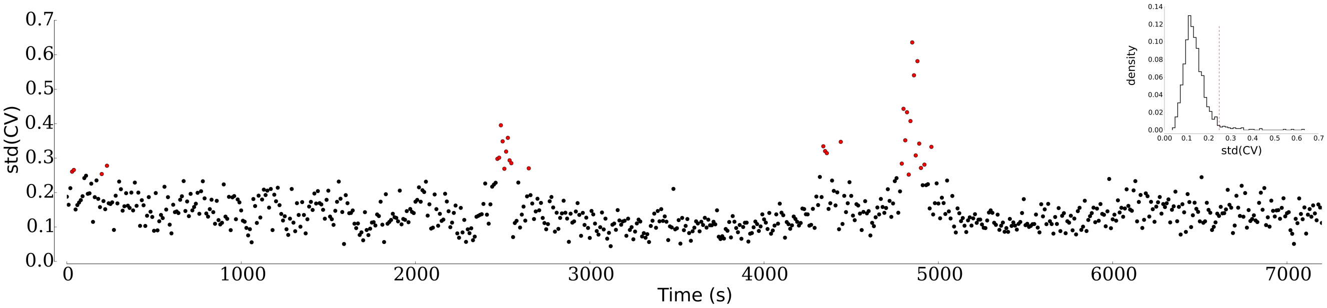

Figure S2. A quantification for the occasional decoupling based on the standard deviation for the coefficient of variation (CV) across local neuronal networks, in each 10s-long period (filled circles), throughout the experiment shown in Figure 1: black value$< 2\sigma$, otherwise in red; 32 circles filled in red imply 320s out of 7200s. Inset shows the distribution of this measure with a vertical red line indicating the arbitrary threshold (2σ).

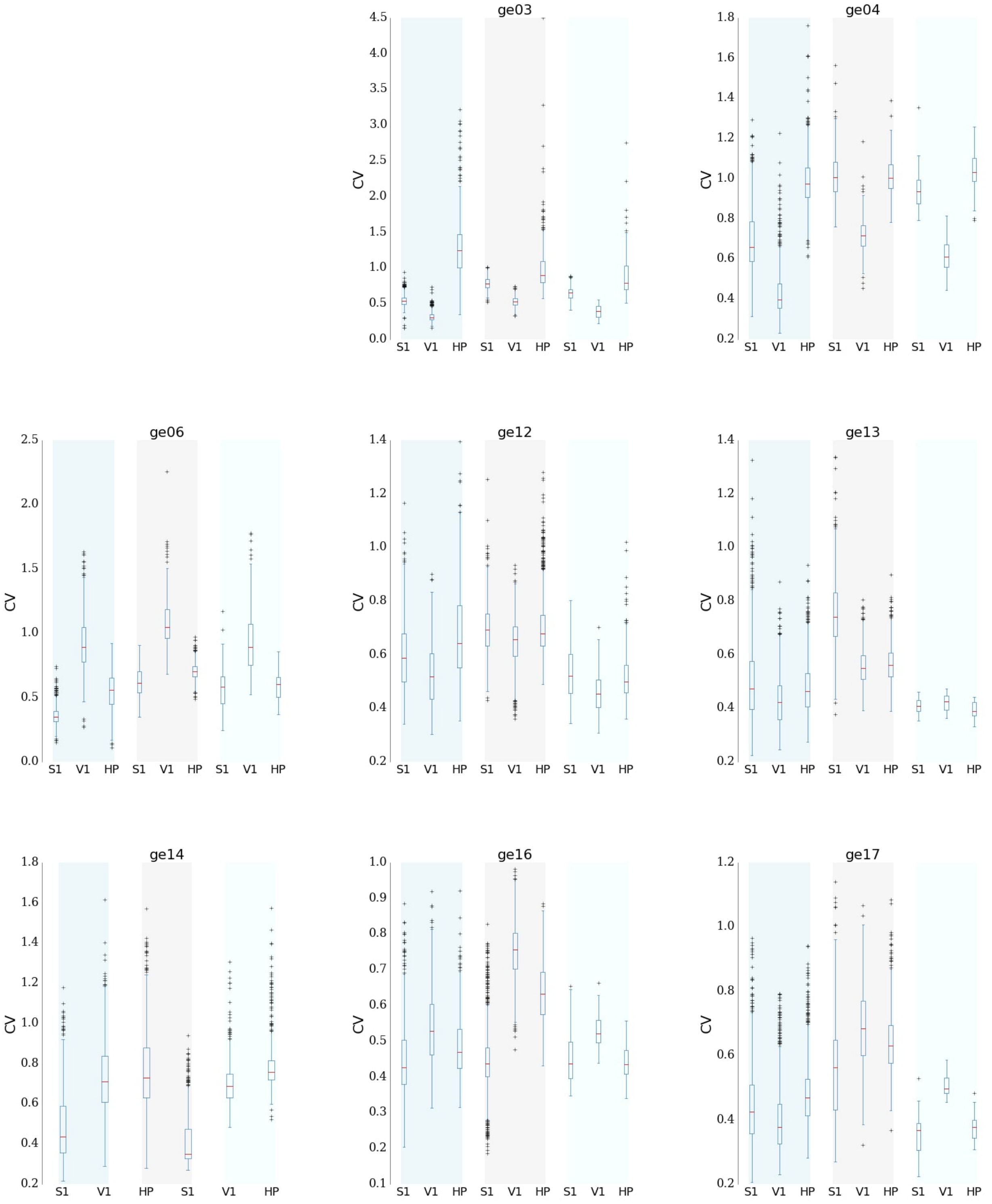

Figure S3. Boxplots for coefficient of variation found in primary sensory neocortex: somatosensory and visual; and hippocampus, where are represented three different behaviour states: wakefulness (WK), slow wave sleep (SWS) and rapid eyes movement (REM); which are plotted by using different colours according the correspondent legend.

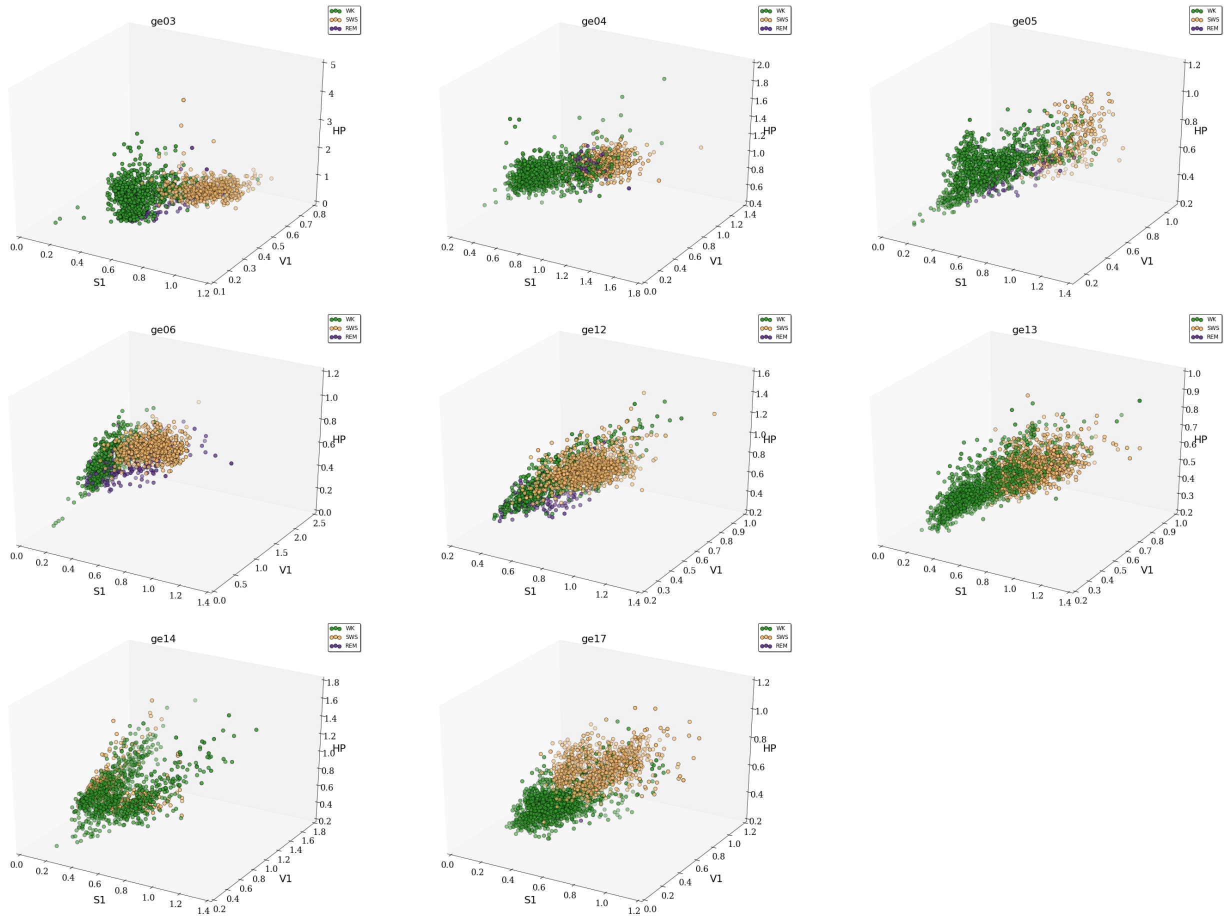

Figure S4. 3D Scatter plots of coefficient of variation found in two areas of primary sensory neocortex: somatosensory (S1) and visual (V1) and hippocampus; for each animal, where are represented three different behaviour states: wakefulness (WK), slow wave sleep (SWS) and rapid eyes movement (REM); which are plotted by using different colors according the correspondent legend, where also can be found the Pearson correlation coefficient. It could not be possible identify REM sleep during recordings from animal ge14.

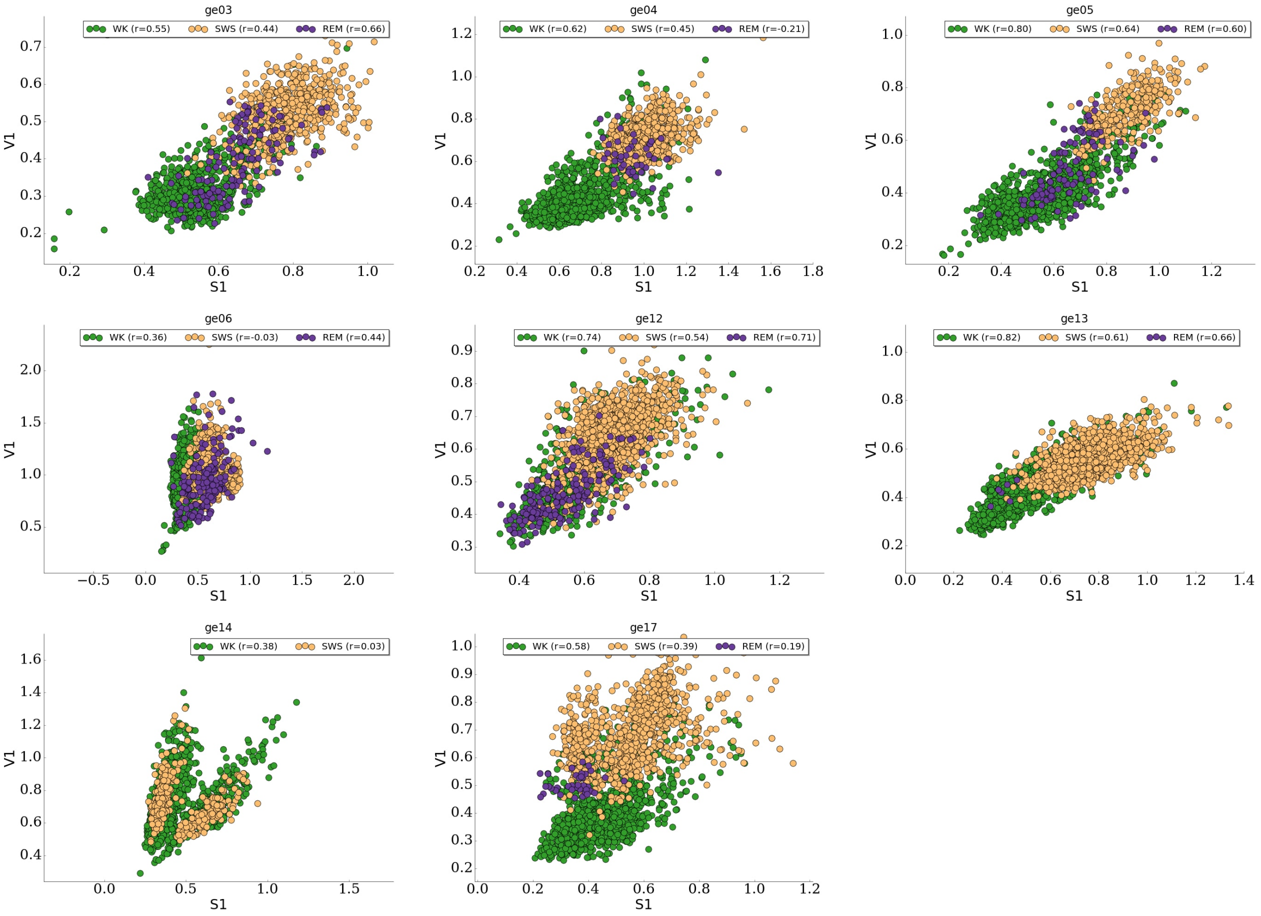

Figure S5. Scatter plots of coefficient of variation found in two areas of primary sensory neocortex: somatosensory (S1) and visual (V1); for each animal, where are represented three different behaviour states: wakefulness (WK), slow wave sleep (SWS) and rapid eyes movement (REM); which are plotted by using different colours according the correspondent legend, where also can be found the Pearson correlation coefficient. It could not be possible identify REM sleep during recordings from animal ge14.

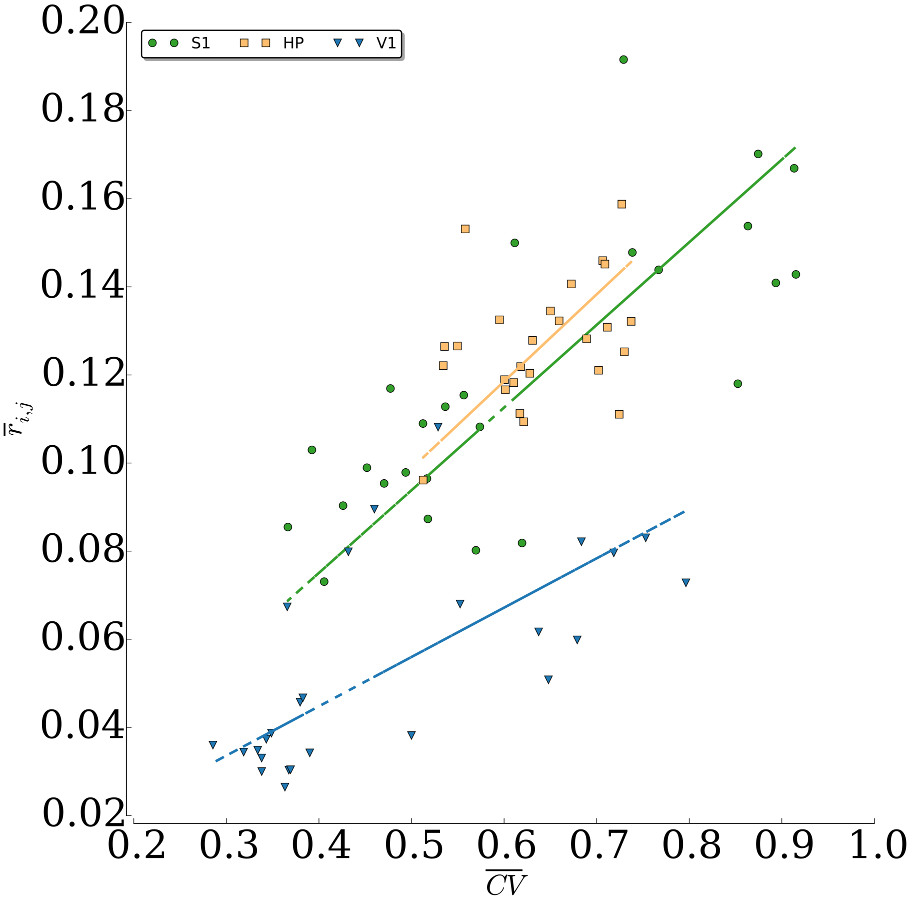

Figure S6. Scatter plot of the average coefficient of variation, $\overline{CV}$, versus mean of the local spiking correlation,$\bar{r}_{i,j}$, in independent 300s-long periods within [300;7700] seconds for the experiment shown in Figure 2. Each CV's sample was based on 10s-long independent time periods, each one binned in time periods of 50ms; the local spiking correlation was calculated based on Mexican hat kernel (T=0.1;J=0.4); each colour used in point clouds and the correspondent regression lines represents one brain area: S1 (green circles, $R^2 = 0.96$), HP (orange squares, $R^2 = 0.98$) and V1 (blue triangles, $R^2 = 0.91$); dashed parts in regression lines indicate low density of samples in the correspondent region.
